# Differences in the amplitude of low‐frequency fluctuation between methamphetamine and heroin use disorder individuals: A resting‐state fMRI study

**DOI:** 10.1002/brb3.1703

**Published:** 2020-07-15

**Authors:** Yan Liu, Jia Zhu, Qiang Li, Yarong Wang, Yongbin Li, Jiajie Chen, Shan Dang, Jing Chen, Hong Shi, Jiuhua Xue, Wei Li, Wei Wang

**Affiliations:** ^1^ Department of Radiology Tangdu Hospital The Air Force Medical University Xi'an China; ^2^ Department of Radiology Xi'an Gem Flower Changqing Hospital Xi'an China; ^3^ Department of Radiology The First Affiliated Hospital of Xi'an Jiaotong University Xi'an China; ^4^ Xi'an No.1 Hospital Xi'an China

**Keywords:** drug use disorder, heroin, methamphetamine, neuroimaging, psychological disorder, resting‐state function

## Abstract

**Introduction:**

Methamphetamine has surpassed heroin as the most popular abused drug in China. Although the use of both heroin and methamphetamine leads to use disorders through dysfunction of the dopamine pathway, the incidence of psychiatric disorder caused by methamphetamine abuse is higher than the incidence of psychiatric disorder caused by heroin abuse. The difference in resting‐state function between heroin use disorder (HUD) and methamphetamine use disorder (MAUD) and the relationship between resting‐state function and psychiatric disorder related to MAUD are unknown.

**Methods:**

In the present study, 21 male individuals with MAUD, 21 demographically matched individuals with HUD, and 21 normal controls (NC) were recruited. The amplitude of low‐frequency fluctuation (ALFF) during resting‐state brain function was measured by magnetic resonance imaging. Psychiatric status was evaluated by the Symptom Checklist‐90 (SCL90).

**Results:**

Individuals with MAUD had increased SCL90 scores compared to those of the NC for anxiety, paranoia, and additional items, and the hostility score was significantly increased compared to that of individuals with HUD. There is no significant difference between HUD and NC individuals. Individuals with MAUD had increased ALFF compared to those of the NC for thalamus, right postcentral, and right inferior temporal gyri, but a decreased ALFF in the cerebellum. Individuals with HUD had significantly increased ALFF compared to those of the NC for left middle frontal gyrus but a decreased ALFF in the left postcentral gyrus. Individuals with MAUD had significantly increased ALFF compared to those of the HUD for thalamus, the right inferior temporal, and bilateral postcentral gyri, and the ALFF of cerebellum and left middle frontal was significantly increased.

**Conclusions:**

Methamphetamine can induce more serious psychiatric disorders than heroin. The resting‐state function involved in mood adjustment, the auditory, and memory‐related brain regions may affect psychotic symptoms related to MAUD.

## Graphical abstract

Methamphetamine can induce more serious psychiatric disorders than heroin. The resting‐state function involved in mood adjustment, auditory, and memory‐related brain regions may affect psychotic symptoms related to methamphetamine use disorder.

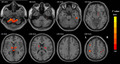

## 1 INTRODUCTION

Drug addiction, the most severe form of drug use disorder, is a chronic brain disorder related to strong biosocial factors that has devastating consequences to individuals and to society (Volkow & Boyle, 2018). Heroin and methamphetamine (MA) are both popular drugs in China. They are considered highly addictive drugs and often cause severe consequences, including mortality, morbidity, and criminality (Hser, Evans, Huang, Brecht, & Li, 2008). According to the Annual Report on Drug Control in China, at the end of 2017, among the 2.553 million registered drug use disorder patients, the number of synthetic drug (mainly MA) abusers has risen to 60.2%, and the number of traditional opioid (mainly heroin) abusers has dropped to 38%. Methamphetamine has surpassed heroin as the most popular abused drug in China.

Both methamphetamine use disorder (MAUD) and heroin use disorder (HUD) are linked to dopamine system dysfunction, and the mental symptoms induced by MAUD and HUD are significantly different (Barr et al., 2006; Volkow, Fowler, Wang, & Goldstein, 2002; Volkow, Fowler, Wang, Swanson, & Telang, 2007). HUD can result in general mental disorder, such as emotional disorder and reduced willpower (Blum et al., 2013), while MAUD can induce schizophrenia‐like symptoms such as paranoia, auditory hallucinations, and insanity, which has been called methamphetamine‐associated psychosis (MAP) (Bramness et al., 2012; Jacobs, Fujii, Schiffman, & Bello, 2008; Lu, Fang, & Wang, 2008). A study by Panenka et al. (2013) reported that in 295 individuals with MAUD, 70% of participants were diagnosed with a mental illness. Previous studies found that although some people's MAP symptoms resolved in <1 month after withdrawal from the drugs, in 30% of people, the MAP symptoms persisted for 6 months, and in 10% of people, the MAP symptoms persisted for longer than 6 months (Deng et al., 2012; Iwanami et al., 1994). In addition, 25%–50% of subjects with long‐term MAUD suffer from MAP during their lifetime (Ipser et al., 2018).

The neurobiology underlying MAP has not been fully discerned. Studies have shown that MAP may be mediated by the frontal, thalamus, limbic, and striatal regions of the brain (Hsieh, Stein, & Howells, 2014). Neuroimaging provides a reliable technical means for studying the neurobiological mechanism of MAP. In vivo human studies using positron emission tomography (PET) and arterial spin labeling (ASL) magnetic resonance imaging, researchers found changes in the parameters of glucose metabolism and cerebral perfusion in the frontal, striatal, and limbic regions of MAUD and psychotic individuals (Vuletic et al., 2018). There are, however, no resting‐state functional MRI data that reveal the neurocircuitry underling MAP. The difference between the influence of MA and heroin on mental symptoms has received widespread attention, but few related studies have been conducted in vivo in humans using neuroimaging methods to compare MAUD and HUD. Alaee, Zarghami, Farnia, Khademloo, and Khoddad (2014) found a higher signal in the white matter of the frontal region of MA abusers' brains compared to that in opiate abusers, and they presumed that the blood flow defects and ischemic lesions in the brain in MA users were greater than in opiate users. Whether the brain regions related to the formation of MAP have different resting‐state functionality between patients with MAUD and HUD was unknown. MAUD had become a serious worldwide public health problem, but there is still a lack of effective treatment. Developing effective methods for intervention and treatment of MAUD will require establishing a clear understanding of the mechanisms underlying this condition.

The SCL‐90 is a self‐report 90‐item scale on which respondents choose one of five descriptive responses for each item that represents the level of severity for that symptom. It contains a wide range of psychiatric symptomatology, from feeling, emotion, thinking, consciousness, behavior to living habits, interpersonal relations, diet and sleep, etc., all of them involve. Ten factors were used to reflect the psychological symptoms of somatization, obsession, interpersonal sensitivity, depression, anxiety, hostility, terror, paranoia, psychosis, and additional items. “Additional items” mainly represent the sum of items not included in the first nine factors, and they mainly reflect sleep and diet (Derogatis & Melisaratos, 1983). In past years, neuroimaging technology has been used to reveal the underlying neural mechanisms of MAUD and HUD development. The specific task‐related fMRI revealed brain responses to external stimuli (Salo, Fassbender, Buonocore, & Ursu, 2013; Wang et al., 2013). However, it is very important to determine the pattern of brain tissue and function in the baseline of the abusers. The amplitude of low‐frequency fluctuation (ALFF) is a biomarker for assessing brain physiological state and calculates the intensity of regional spontaneous neuronal activity as the square root of the power spectrum in a low‐frequency range (Zang et al., 2007). The abnormal neuronal activity during resting state may serve as an adequate marker to reflect the progress and impaired executive function of multiple brain diseases (Yuan et al., 2016, 2017). ALFF is a research method to evaluate the amplitude of each voxel from the perspective of energy and reflect the level of spontaneous activity of neurons in resting state (Zang et al., 2007). In order to improve the normality of ALFF, Z‐transform is used, that is, subtracting the whole‐brain average signal. Those ALFF values that are lower than the average brain signal become negative. The higher ALFF within brain areas may reflect higher spontaneous neuronal activity during resting. The ALFF method has been widely used in addiction research (Jiang et al., 2011; Wang et al., 2013).

In this study, we combined resting‐state fMRI with psychological assessment to observe the differences between individuals with MAUD and HUD to explore the correlation between brain regions and psychological symptoms. To look at combined imaging techniques and psychological scaling methods may provide insights into the neural mechanisms underlying MAP, and hope to provide help for the development of effective drug treatment strategy and the selection of surgical target.

## 2 MATERIALS AND METHODS

### 2.1 Participants

A total of 27 individuals with MAUD were recruited from the community in Xi'an, China; Six patients were excluded due to head movement (4/6), structural abnormality (1/6), and unable to complete the image acquisition (1/6). Therefore, 21 individuals with MAUD were included in this study. Twenty‐one demographically matched individuals with HUD and 21 NC were recruited from the same community. More detailed demographic information is given in Table 1.

**TABLE 1:** Demographic information of participants

Characteristics	MAUD (*n* = 21)	HUD (*n* = 21)	NC (*n* = 21)	*F* value	*p* value
Age (years)	28.3 ± 5.2	31.4 ± 7.6	31.5 ± 7.0	1.454	.242
Education level (years)	9.4 ± 2.1	10.7 ± 2.9	10.6 ± 2.2	1.745	.183
Cigarettes (per day)	19.9 ± 9.7	17.9 ± 6.4	14.1 ± 6.8	3.010	.057
Duration of drug use (months)	29.1 ± 18.7	42.9 ± 25.5	NA	3.974	.053

Note

*p* value for the statistical tests: one‐way ANOVA for age, education, and number of cigarettes (across three groups) and two‐sample *t* tests for the drug use measures (MAUD vs. HUD).

Abbreviations: HUD, heroin use disorder; MAUD, methamphetamine use disorder; NC, normal controls.

The inclusion criteria for the individuals with MAUD and HUD required the participants to (a) meet the diagnostic criteria for substance use disorder in the DSM‐V; (b) have a heroin or MA use history longer than 12 months; (c) have no history of mixed drug use; (d) be aged 18–50 years; and (e) be right‐handed, as judged by the Edinburgh Handedness Inventory. Individuals were excluded if they had (a) any history of head trauma and/or neurological disease or neurological disease signs; (b) daily alcohol consumption; (c) current medical illness; or (d) claustrophobia or any contraindication for MRI examination.

This study was approved by the Institutional Review Board of the Tangdu Hospital, Air Force Medical University, Xi'an, China. All participants were fully informed of the details and aims of the study and the experiment and provided written consent for their involvement.

### 2.2 Psychiatric status evaluation

The Symptom Checklist‐90 (SCL‐90) was used for the self‐reported psychiatric‐state evaluation of all participants under the guidance of physicians before the magnetic resonance imaging scan.

### 2.3 Image acquisition

All MRI data were acquired on a 3 T MRI scanner (GE Signa Excite HD) using an eight‐channel head coil. Subjects lay supine with their heads fixed by a belt and foam pads and were instructed to keep their heads still, to open their eyes and stare at the “+” sign, and not to think of anything specific. Before the formal fMRI scan, the subjects underwent a mock scan for 1 min to familiarize themselves with the scanning environment. A routine T2WI MRI scan was conducted to exclude individuals with gross structural abnormalities. Then, the functional images were acquired using a gradient echo planar imaging sequence with the following settings: 32 axial slices, TR 2,000 ms, TE 30 ms, flip angle 90°, spatial resolution 4 × 4 × 4 mm^3^, slice thickness 4.0 mm, FOV 256 × 256 mm^2^, and matrix 64 × 64. Then, a high‐resolution fast spoiled gradient echo image was also collected with the following parameters: TR 7.8 ms, TE 3.0 ms, slice thickness 1.0 mm, FOV 256 × 256 mm^2^, matrix 256 × 256, and spatial resolution 1 × 1 × 1 mm^3^. After scanning, all subjects reported that they were awake during all scans.

### 2.4 Data analysis

The differences in age, education level, and smoking status were assessed by one‐way analysis of covariance (ANOVA), and the difference in drug use duration was assessed by independent sample *t* test; *p* < .05 was considered significant. The imaging data were analysis with SPM8 (http://www.fil.ion.ucl.ac.uk/spm) and DPABI (http://rfmri.org/dpabi) software. Image data were slice time corrected, motion corrected, and normalized to a standard SPM T1 template. The samples were interpolated to 3‐mm isotropic voxels and then spatially smoothed with an isotropic 8‐mm full‐width half‐maximum Gaussian kernel. In order to remove the effects of global, white matter, cerebrospinal fluid signals, and head motion on the results, six head motion parameters and mean time series of global, white matter, and cerebrospinal fluid signals were included as covariates into a random effect model. Subjects with excessive head motion (more than 1.5 mm in translation or 1.5° in rotation) were excluded from the analysis. After bandpass filtering (0.01–0.08 Hz) and linear‐trend removal, the time series were transformed to the frequency domain using a fast Fourier transform, and the power spectrum was obtained. Since the power of a given frequency is proportional to the square of its amplitude in the original time series, the square root was calculated at each frequency of the power spectrum and then averaged across 0.01–0.08 Hz to yield a measure of ALFF from each voxel. The ALFF was calculated by averaging the values of each region of interest (ROI). ROI is defined based on the brain regions with significant differences among the three groups of subjects. The center point of ROI is the coordinate peak of the different brain regions, and 3 mm is the spherical radius to define ROI. Differences in ALFF were examined with multiple comparisons using threshold‐free cluster enhancement (TFCE) at the whole‐brain level among the MAUD, HUD, and NC group at single voxel‐level threshold of *p* < .001; the results corrected with family‐wise error (FWE).

Pearson correlation analysis was performed to explore the relationship between the ALFF in identified brain regions and the psychological scores in the MAUD group; *p* < .05 was considered significant.

## 3 RESULTS

### 3.1 Psychiatric status

The anxiety, hostility, and paranoia items of the SCL‐90 showed significant differences among the three groups. Compared with the NC group, the MAUD group showed higher levels of anxiety, paranoia, and additional items, while no significant difference was found between the HUD and NC groups. Compared with the HUD group, the MAUD group had higher scores for hostility. The psychological scores are summarized in Table 2.

**TABLE 2:** SCL‐90 scores and two‐by‐two post hoc comparison results among the three groups (*p* < .05, Bonferroni correction)

Items	Mean ± *SD*			*p* value from ANOVA	Post hoc		
	MAUD	HUD	NC		MAUD versus NC	HUD versus NC	MAUD versus HUD
Somatization	0.68 ± 0.69	0.44 ± 0.31	0.38 ± 0.50	.162	0.21	1	0.46
Obsessive–compulsive	0.95 ± 0.76	0.58 ± 0.33	0.57 ± 0.63	.078	0.14	1	0.16
Interpersonal sensitivity	0.86 ± 0.80	0.59 ± 0.41	0.47 ± 0.60	.124	0.14	1	0.48
Depression	0.83 ± 0.81	0.75 ± 0.49	0.45 ± 0.57	.140	0.18	0.40	1
Anxiety	0.75 ± 0.71	0.52 ± 0.37	0.30 ± 0.41	.027*	0.02^a^	0.53	0.49
Hostility	1.25 ± 1.05	0.52 ± 0.53	0.62 ± 0.85	.014*	0.06	1	0.02^b^
Terror	0.42 ± 0.60	0.27 ± 0.26	0.22 ± 0.32	.279	0.38	1	0.72
Paranoia	0.92 ± 0.96	0.42 ± 0.38	0.41 ± 0.52	.023*	0.04^a^	1	0.06
Psychoticism	0.60 ± 0.62	0.48 ± 0.34	0.42 ± 0.48	.501	0.75	1	1
Addition item	0.87 ± 0.67	0.75 ± 0.44	0.43 ± 0.52	.032*	0.03^a^	0.20	1

Abbreviations: HUD, heroin use disorder; MAUD, methamphetamine use disorder; NC, normal controls.

^a^ NC < MAUD.

^b^ HUD < MAUD.

* Significant difference (*p* < .05).

### 3.2 Imaging

**FIGURE 1:**
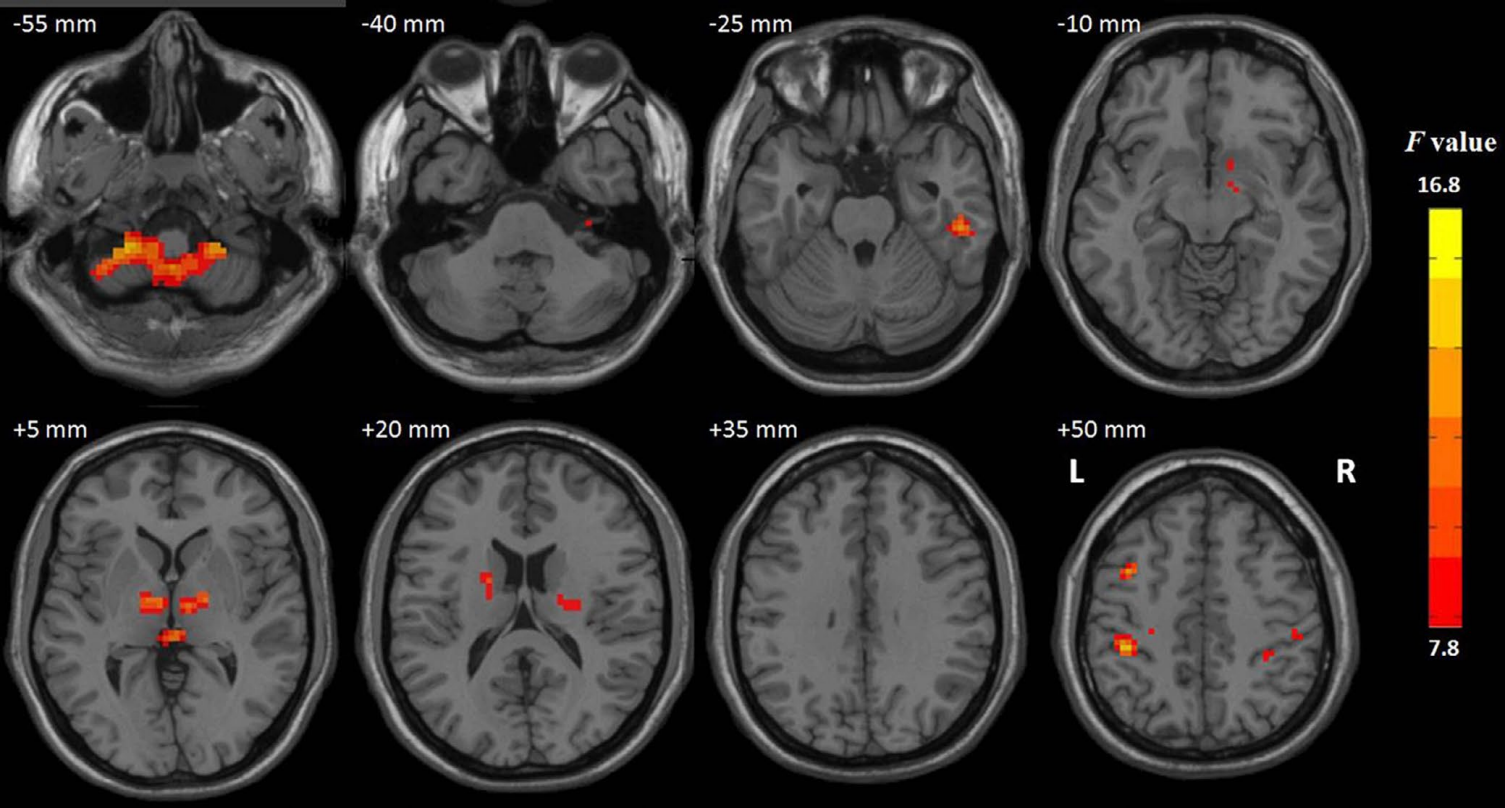
Red indicates that the three groups had significant ALFF differences (*p* < .001, TFCE corrected). L, left; R, right

**FIGURE 2:**
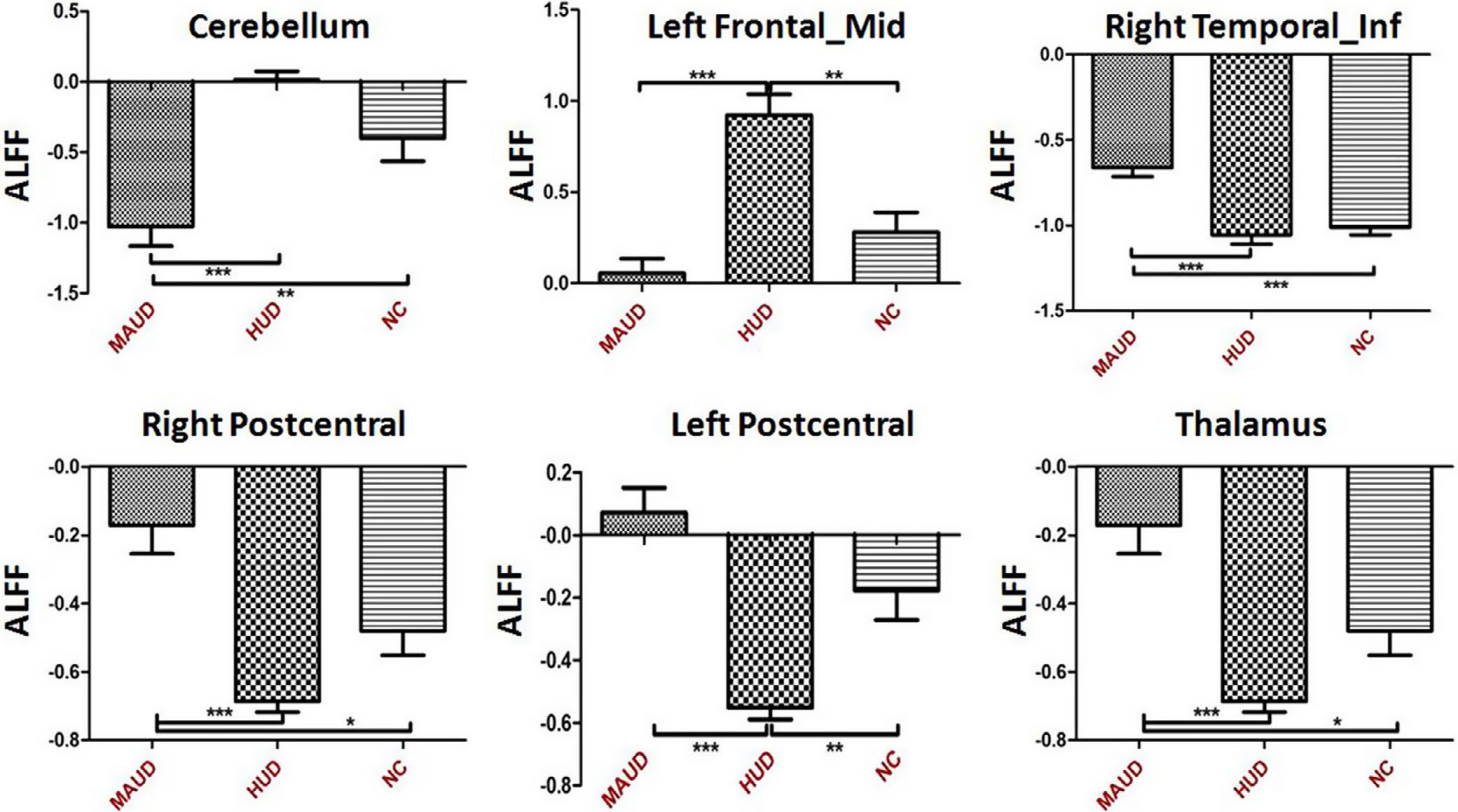
Differences in ALFF among the three groups by brain region. HUD, heroin use disorder; MAUD, methamphetamine use disorder; NC, normal controls. Significant difference: *p < .05; **p < .01; ***p < .001

The ALFF values showed significant differences in the cerebellum, thalamus, left middle frontal gyrus, right inferior temporal gyrus, and bilateral postcentral gyrus among the three groups. More details are provided in Table 3. Compared with the NC group, the MAUD group demonstrated a significantly increased ALFF in the thalamus and the right postcentral and right inferior temporal gyri, but a decreased ALFF in the cerebellum. Compared with the NC group, the HUD group demonstrated a significantly increased ALFF in the left middle frontal gyrus but a decreased ALFF in the left postcentral gyrus. Compared with the HUD group, the MAUD group demonstrated significantly decreased ALFF in the cerebellum and left middle frontal gyrus but increased ALFF in the thalamus and the right inferior temporal and bilateral postcentral gyri (Figures 1 and 2).

**TABLE 3:** The clusters of significant differences among brain regions are shown in three groups

Location	Voxel size	Talairach coordinates			*F* value
		*X*	*Y*	*Z*	
Cerebellum	373	−21	−39	−57	16.51
Left frontal_mid	57	−36	12	54	19.10
Left postcentral	186	−33	−21	57	17.76
Right postcentral	159	36	−18	57	15.58
Right temporal_inf	117	51	−24	−27	18.53
Thalamus	265	3	−27	6	15.58

### 3.3 Correlation results

**FIGURE 3:**
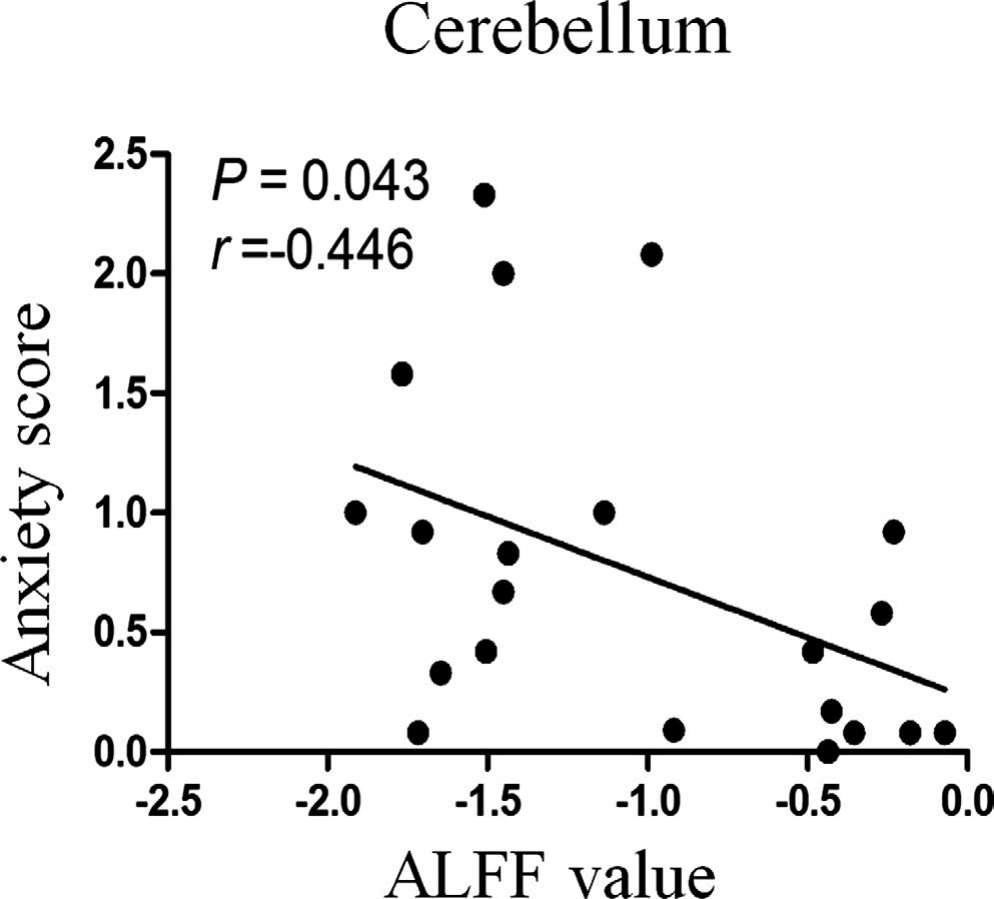
Bivariate scatter plot. A negative correlation between anxiety score and ALFF in the cerebellum of the methamphetamine use disorder group

Before multiple comparison correction, the ALFF value of the cerebellum was negatively correlated with the anxiety score in the MAUD group (*r* = −.446, *p* = .043) (Figure 3). But after multiple comparison correction, the correlation did not survive. Other correlation results are provided in the supplementary.

## 4 DISCUSSION

To the best of our knowledge, this is the first neuroimaging study to explore the brain function differences between individuals with MAUD or HUD in the resting state. The main findings of this study were as follows: (a) MAUD could cause multiple positive mental symptoms, including anxiety, hostility, and paranoia, and the MAUD group had higher scores for hostility than the HUD group; (b) compared to the HUD group, the MAUD group had increased brain activity in the thalamus, right inferior temporal gyrus, and bilateral postcentral gyrus, and decreased brain activity in the cerebellum and left middle frontal gyrus; and (c) there was no significant correlation between different brain regions and mental scale.

The results from the ALFF analysis are in line with previous findings about methamphetamine addiction and indicate that the frontal lobes and thalamus were changed, possibly due to decreased blood flow and metabolism in these areas (Hsieh et al., 2014; Vuletic et al., 2018). Deficits in frontal lobe structure have been widely reported in MA users (London, Kohno, Morales, & Ballard, 2015). The prefrontal cortex, an important part of the brain associated with addiction, is related to motivation‐driving and inhibitory control (Baler & Volkow, 2006; Volkow et al., 2002). The middle frontal gyrus is considered to be mainly responsible for working memory and motivation, and its increased activity is believed to be related to the higher desire for drugs and frequent impulses to find and use drugs (Li et al., 2015; Wang et al., 2013). In this study, compared with the NC and MAUD groups, the HUD group demonstrated significantly increased brain activity in left middle frontal gyrus. It may be an important reason that individuals with HUD have more serious physical dependence, easily leading to addiction and stronger cravings for the drug.

In addition, the frontal lobe can also produce auditory hallucination through the principle of uninhibited excitability of auditory centers and through its effect on the efficient network connectivity of auditory language perception (Mulert, Kirsch, Pascual‐Marqui, McCarley, & Spencer, 2011; Plaze et al., 2006). The temporal lobe is the site of the auditory language center, and the activity of the temporal lobe and auditory‐related cortex (frontal lobe, parietal lobe, and limbic system) in auditory hallucinatory patients can be detected (Wang, Metzak, & Woodward, 2011). Researchers found that frontal lobe function decreased and temporal lobe function increased, contributing to auditory hallucination, in patients with schizophrenia (Ćurčić‐Blake et al., 2017). Our findings show a similar pattern of decreased activity in the left frontal middle gyrus and increased activity in the right inferior temporal gyrus in the MAUD group compared to the HUD group. Auditory hallucination is a common symptom of MAP, with a higher frequency of auditory hallucination of up to 48.5% found in acute MAP patients (Shelly et al., 2016). Although we did not perform a specialized auditory hallucination assessment, we found that individuals with MAUD have a higher frequency and degree from which to choose options about auditory hallucinations in the SCL‐90. Therefore, we presumed that decreased activity in the frontal middle gyrus and increased activity in the temporal gyrus may play a role in the higher incidence of auditory hallucination in individuals with MAUD.

The cerebellum was shown to have significantly decreased activity in the MAUD group compared to the HUD and NC groups. Although there is no significant correlation between cerebellar activity and anxiety scores, they have a negative correlation trend in the MAUD group. In addition to motor function, the cerebellum is involved in mood regulation, cognition regulation, and memory processes. People with cerebellar injury showed deficits in learning, memory and cognitive function (Carbo‐Gas et al., 2014; Nestor, Ghahremani, Monterosso, & London, 2011). The cerebellum can form feed forward circuits through the thalamus, which plays a role in cognitive and emotional processing. The cerebellum and frontal lobe are linked to higher cognitive activity in humans (Middleton & Strick, 2001). Research has found that cerebellar vermis abnormalities can lead to emotional disorders, social dysfunction, and autism (Rüsch et al., 2007). Our results showed that decreased cerebellum activity was closely related to higher anxiety scores. However, more fMRI studies are needed to understand the emotional regulatory role of the cerebellum in individuals with MAUD.

The activity of the thalamus in the MAUD group was significantly higher than in the HUD group in our study. The thalamus is considered to act as a “relay station” by filtering and gating sensory inputs to the cerebral cortex. It was found that the group of anterior thalamic nuclei could be related to the anterior cingulate gyrus and prefrontal cortex (Järvenpää et al., 2018; Weininger et al., 2019). The ventrolateral thalamus is connected with the upper frontal cortex and the prefrontal cortex. The damage of ventrolateral thalamus can cause extensive damage to the attention circuit (de Bourbon‐Teles et al., 2014). Moreover, the thalamus receives projections from the accumbens nucleus and the orbital frontal cortex. It also sends projections back to these regions, forming the striato‐thalamo‐orbitofrontal (STO) circuit through which the reinforcing responses to abuse drugs can be modulated (Volkow, Fowler, & Wang, 2004). In addition, the loss or metabolic changes of neurons in the dorsomedial thalamic nucleus may lead to schizophrenia, which is mainly caused by the dense excitability interaction of neurons between dorsomedial thalamic nucleus and prefrontal cortex (Ouhaz, Ba‐M'hamed, Mitchell, Elidrissi, & Bennis, 2015). To justify our findings, we speculate that a compensation mechanism exists: MAUD individuals with positive mental symptoms may have increased thalamic activity to compensate for decreased activity of the prefrontal cortex, which is functionally connected to the thalamus. The postcentral gyrus serves the mirror neuron system and is closely related to empathy and emotional expression (Kohler et al., 2002). The impaired postcentral gyrus may be one of the neural mechanisms for aggressive behavior (Kumari et al., 2006). An increase in ALFF value in the bilateral postcentral gyrus and in the hostility score of the MAUD group compared to that of the HUD group was shown in this study and is in line with work by Tiihonen et al., who showed that decreased volume of gray matter in the postcentral gyrus was associated with violent tendencies in schizophrenia and mental disorders (Tiihonen et al., 2008).

In the current study, differences were observed in various brain regions between the MAUD and HUD groups, and the alteration in these brain regions may play an important role underling the distinction of the mental symptoms. However, several limitations should be mentioned. First, the psychological scales are not sufficiently comprehensive. The self‐reporting scale used in the current study is not as accurate as other rating scales because it can be affected by the mood of the patients at test time. Second, all participants were males, and the effects of MA on sex cannot be assessed. Finally, Kashyap, Bhattacharjee, Yeo, and Chen (2020) have shown that the general categorization of subjects based only on external symptoms (e.g., healthy vs. diseased, control vs. patient) should also consider aspects of a healthy subject's lifestyle habits and psyche. We did not select the control group from open datasets, which would bring some bias to the results (Kashyap et al., 2020).

## 5 CONCLUSION

This is the first study to explore the local neuronal activity in MAUD patients compared to NC and HUD patients using a resting‐state fMRI study combined with a self‐reported mental health assessment. We demonstrate the significant differences in ALFF in regions related to mood adjustment, auditory processing, and memory in MAUD patients, which may help shed some light on the neurobiological mechanisms underlying MAUD‐associated psychosis. These findings are helpful for understanding the different clinical manifestations between MAUD and HUD patients and provide a reliable theoretical basis for the clinical treatment of MAP.

## CONFLICT OF INTEREST

The authors certify that they have no commercial associations that might pose a conflict of interest in connection with this article.

## AUTHORS' CONTRIBUTIONS

YL, JZ, WW, and WL were responsible for the study design. SD, JC, HS, and JX contributed to the acquisition of MRI and demographic data. YL, JC, and QL performed the data analysis. WL, WW, and YW assisted with data analysis and interpretation of findings. YL and WL drafted the manuscript. All authors critically reviewed the content of the manuscript and approved the final version for publication.

## Data Availability

The data that support the findings of this study are available from the corresponding author upon reasonable request. However, due to regulations, we are not able to share the fMRI files.
